# DES Selection for Left Main and Coronary Bifurcation Stenting

**DOI:** 10.31083/j.rcm2409266

**Published:** 2023-09-21

**Authors:** Zlatko Mehmedbegović, Dario Jelić, Đorđe Mladenović, Goran Stanković

**Affiliations:** ^1^Department of Cardiology, University Clinical Center of Serbia, 11000 Belgrade, Serbia; ^2^Faculty of Medicine, University of Belgrade, 11000 Belgrade, Serbia

**Keywords:** drug-eluting stents, bifurcation stenting, left main coronary artery, device selection, device bench testing

## Abstract

Coronary bifurcation lesions present a challenging lesion subset regarding procedural complexity and worse patient outcomes as compared to simple lesions. Drug eluting stents (DES), as the current standard of care for percutaneous myocardial revascularization, have tubular design and uniform diameter, and therefore, need to be subjected to a standardized set of procedural modifications, to optimally fit and reconstruct underlying bifurcation anatomy. Since contemporary DES have various design platforms, with diverse mechanical properties, we must be aware of the device’s favorable characteristics and limitations, to ensure maximal procedural safety and success. This is especially true for bifurcation lesion stenting, during which device integrity will often be eventually tested by undergoing specific procedural steps, such as proximal balloon optimization, kissing-balloon inflations, or even intentional stent crushing. In this review we address the design characteristics of contemporary DES, their bifurcation-specific experimental testing data, and reported clinical results, in an attempt to provide relevant information and help in device selection for bifurcation stenting procedures.

## 1. Introduction

Coronary bifurcation lesions are common and are associated with higher risks of major cardiac events and restenosis after percutaneous coronary intervention (PCI) compared to simple lesions [1, 2]. Due to unique fractal anatomy of coronary bifurcations, their treatment requires understanding of not only a lesion characteristic and tailored stenting strategy, but also of the stent design properties [3, 4]. Drug eluting stents (DES), which have a tubular design, are currently the standard of care for percutaneous revascularization of left-main (LM), as well as a non-left main (non-LM) bifurcation lesions [5, 6]. Due to the step difference in reference lumen diameters within a bifurcation segment, two most important technical aspects determine the achievement of optimal procedural result of bifurcation stenting and correlate with the improved clinical outcomes: (a) maximal stent expansion capacity to match the proximal main vessel (MV) diameter and achieve optimal stent apposition; and (b) ease of subsequent side branch (SB) access, in case of emerging SB compromise and need for further SB intervention. Firstly, as a proximal stent post-dilatation is nowadays a mandatory step of bifurcation PCI, typically with large over-expansion in the setting of large vessel discrepancy, it is recognized that stent platform designs have a critical impact on the achieved over-expansion results [3, 7, 8]. Secondly, to maximize the SB access, a single stent cell needs to be expanded by balloon inflation following MV stenting, additionally emphasizing the role of DES platform design and its impact on both, the maximal expansion capacity and ability to widen the side-cell towards the SB [9]. Further to this, certain bifurcation PCI techniques, like the crush-stenting, involve an intentional physical damage to stent structural integrity, creating layers of deranged metal, double-layers, or even triple-layers [10].

**Fig. 1.**
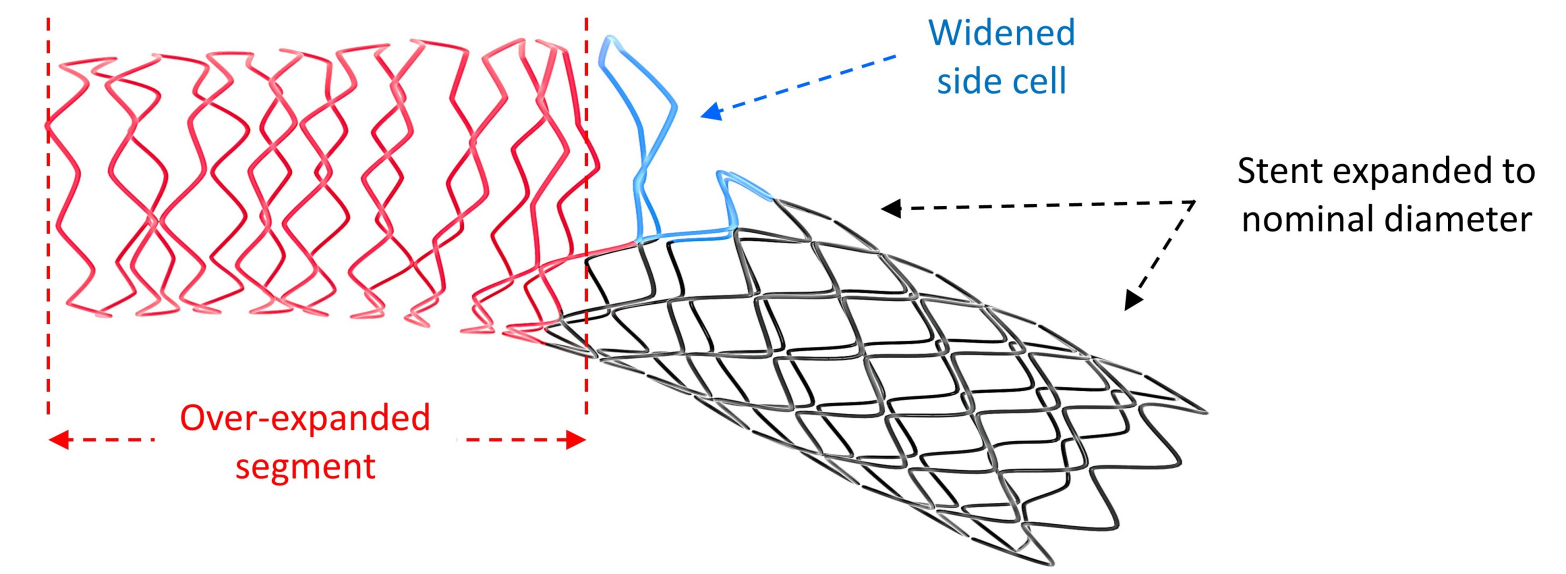
**Illustration of optimized (bifurcation adjusted) stent configuration showing three distinctive regions following standard set of procedural stent modifications**. Red coloured struts illustrate over-expanded proximal segment; Blue coloured struts delineate widened cell at the side branch opening; Black coloured struts at the distal main branch segment of the stent expanded at nominal diameter.

Therefore, to achieve an optimal procedural result of bifurcation PCI, it is essential to understand DES platform design characteristics, focusing on a specific performance property, such as maximal over-expansion capacity and stent cell expansion ability. Since some of this important information is not routinely provided aside from compliance charts and burst pressure data, but comes from *in-vitro* (bench) or virtual (simulation) experiments, the operator must be aware of it beforehand to select the appropriate DES that can withstand necessary modifications during an attempt to optimally reconstruct natural fractal bifurcation anatomy (Fig. 1).

The aim of this review is to comprehend the technical features of modern DES platforms with a focus on device behavior, relative to variations in stent design and different mechanical properties, thereby providing relevant information for device selection, during bifurcation PCI.

## 2. Stent Design Nomenclature

**Fig. 2.**
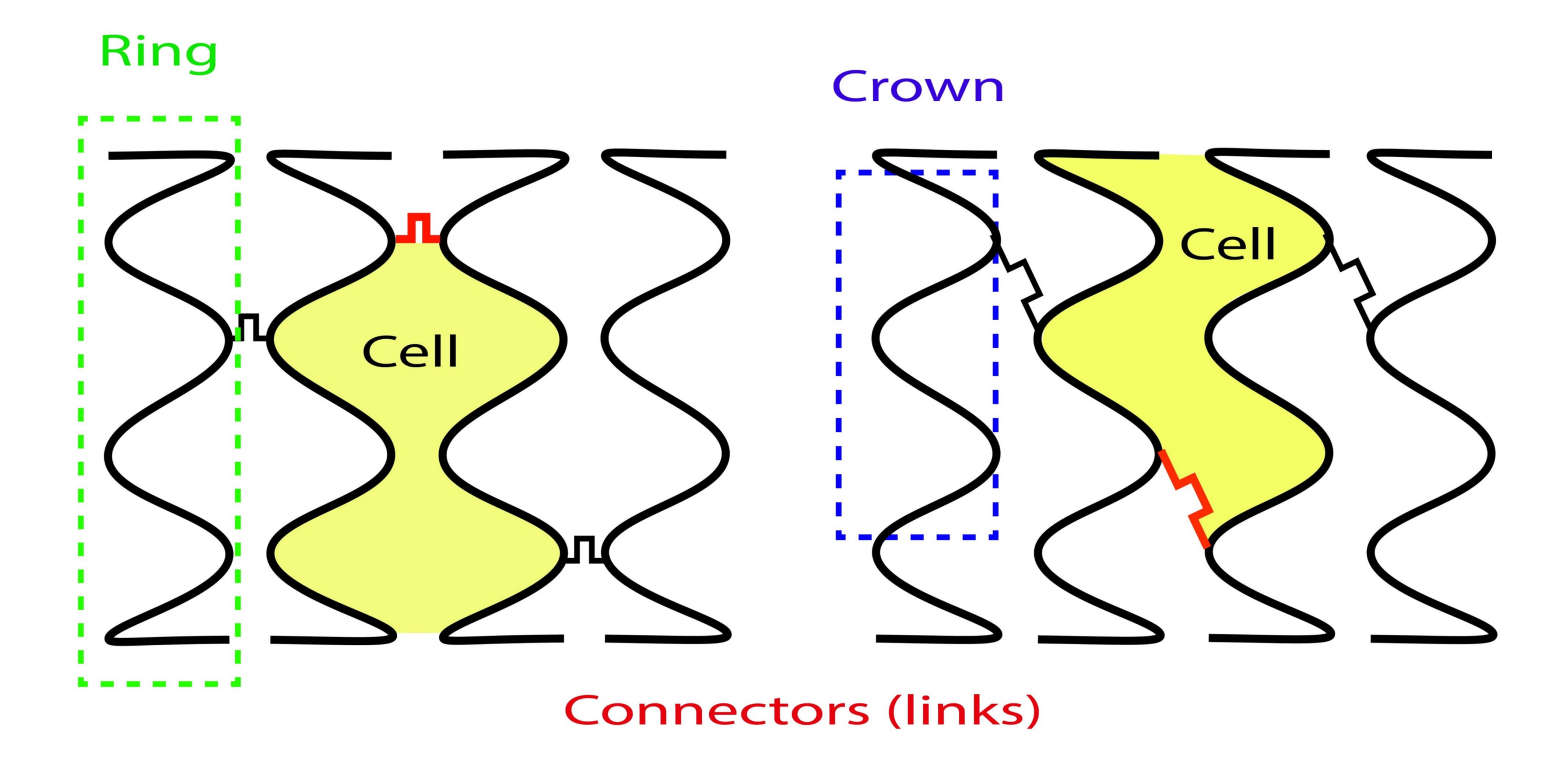
**Stent design nomenclature**. Left panel — Peak to peak design; Right panel — Peak to valley design. Green dotted rectangle showing stent ring comprised of serially connected stent crowns (blue dotted rectangle). Crown consists of two adjacent struts forming an angle. Stent cell (yellow coloured area) is area enclosed by an adjacent connector (red angulated line) and bridged crowns.

Each stent consists of following segments: crown, connectors, ring, and cell (Fig. 2) [3]. Crown or peak is defined as 2 adjacent struts forming an angle. A complete stent ring is formed by a few adjacent serially connected crowns which allow the stent to expand with elongation from a crimped state. Connectors join parallel rings longitudinally. Stent cell is a window area enclosed by an adjacent connectors and crowns. Depending on the offset of peaks in adjacent rings, stent can have two basic configurations. Commonly, DES platforms are made in two or three size designs, named small, intermediate and large vessel model designs, with dividing size around 3 mm, which needs to emphasized, because it dictates the expansion and especially over-expansion capacity.

## 3. Contemporary Drug-Eluting Stent Characteristics

Since the first iteration of DES, numerous stent design advancements that followed, improved their overall safety, procedural and device success, as well as clinical outcomes [11]. The latest generation of DES has excellent safety and efficacy profiles achieved by minimizing the strut thickness, improved deliverability, and either biocompatible or absorbable polymers [12]. DES is constructed by a variety of methods, which ultimately determines their design and physical characteristics as presented in Table 1. With metallic platform made of biocompatible metals like cobalt, platinum, chrome, nickel, etc., superior radio-opacity and higher radial strength were achieved that enabled precise positioning and larger expansion capacities [13]. Both features are of extreme importance during bifurcation stenting, as in case of T- or T and protrusion stenting, when no or minimal protrusion needs to be achieved, or in case when stent needs to be expanded beyond its labeled limits to accommodate the diameter of the MV. The majority of modern DES have reduced strut thicknesses between 60 and 80 μm, as opposed to the 120 to 140 μm of the earlier devices. Thinner struts are advantageous because they decrease the outer and increase stent’s inner diameter, increase its flexibility, and lessen the amount of vascular damage they cause when they are implanted. Clinically, this has corresponded with a decrease in restenosis rates, faster endothelization, less stent thrombosis and improved deliverability with newer metallic platforms [14].

**Table 1.** **Contemporary drug-eluting stents characteristics and bench testing data**.

	Orsiro	Promus Premier	Resolute Onyx	Ultimaster	Xience Sierra	Synergy	Megatron	Relevance for bifurcation PCI
Stent design (4-ring segment illustration)	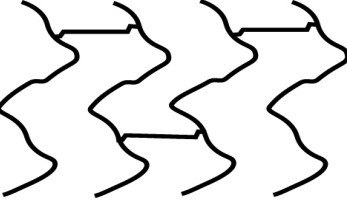	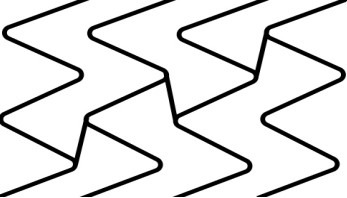	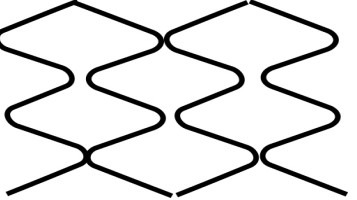	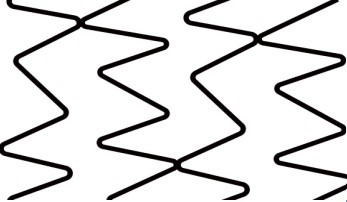	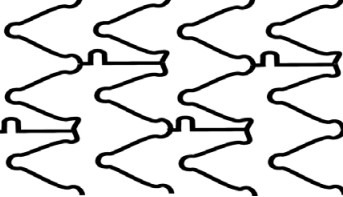	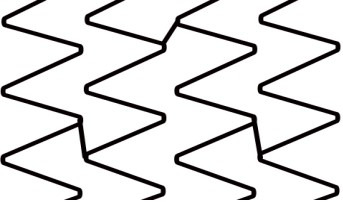	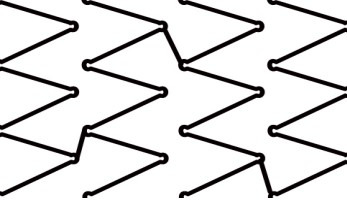	
Stent manufacturing	Laser cut slotted tube	Laser cut slotted tube	Single strained core wire	Laser cut slotted tube	Laser cut slotted tube	Laser cut slotted tube	Laser cut slotted tube	Impact on stent design, and physical properties like flexibility, radial force etc.
Metal	CoCr	PtCr	CoCr	CoCr	CoCr	PtCr	PtCr	Impact on visibility, recoil resistance, tissue inflammation etc.
Strut thickness & shape	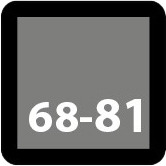	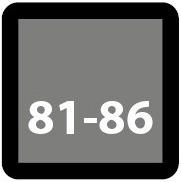	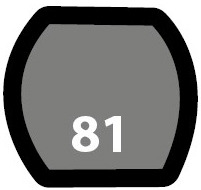	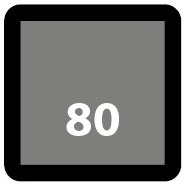	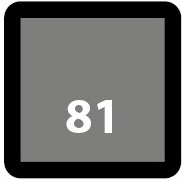	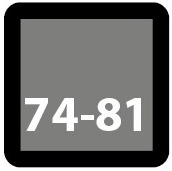	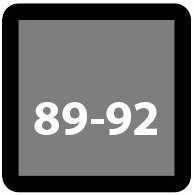	Impact on visibility under fluoroscopy, radial strength, device crosability, SB obstruction
Polymer degradation	Bioresorbable	Permanent	Permanent	Bioresorbable	Permanent	Bioresorbable	Bioresorbable	Impact on drug delivery and inflammation
Polymer thickness	7.4	8	12	15	16	4	4	Impact on final stent crossing profile and final inner stent area
Antirestenotic drug	Everolimus	Everolimus	Zotarolimus	Sirolimus	Everolimus	Everolimus	Everolimus	Antirestenotic properties
Polymer coating distribution	Circumferential	Circumferential	Circumferential	Abluminal	Circumferential	Abluminal	Abluminal	Ablumnial coating only reduces downstream exposure to drug
Available sizes (mm)	2.25–4.0	2.25–4.0	2.0–5.0	2.25–4.0	2.0–4.0	2.25–4.0	3.5–5.0	Availability to accomodate all vessel sizes
Number of rings (per 15 mm device)	6–7	8	10	8	6	8–10	Ø	Impacts flexibility and longitudinal strength
Number of connectors per ring	3–4	2	2–3	2	3	2 in shaft; 4–5 in proximal end	3 in shaft; 4 in proximal 2 rings	Impact on radial force, jailed SB strut cell dilatation
Distal device profile:								
- shaft (French)	2.7	2.7	2.7 (3.2 for 4.50–5.00 mm)	2.7	2.7	2.7	Ø	Important for stent deliverability and crossability, especially for two stent techniques
- lesion entry (inches)	0.017	0.018	0.017	0.017	0.017	0.017	Ø	
- crossing (inches)	0.04	0.04	0.04	0.04	0.039	0.039	Ø	
Labeled maximal over-expansion inner diameter for ≥3.5 mm devices (mm)	4.5	4.25 (3.5 mm stent); 5.75 (4.0 mm stent)	5.75 (≥4.5 mm stent); 4.75 (3.5–4.0 mm stent)	5.5	5.5	5.75 (≥4.0 mm stent); 4.25 (3.5 mm stent)	6	Important for optimal proximal MV stent apposition, especially for large or LM artery
Bench testing data regarding over-expansion ability and SB side cell access and dilatability:								
Meassured average diameter after over-expansion (%) with 6 mm balloon for 4 mm stent [4]	5.3 mm (58%)	Ø	5.6 mm (39%)	5.8 mm (63%)	5.6 mm (67%)	5.7 mm (56%)	Ø	Important for optimal proximal MV stent apposition, especially for large or LM artery
Circular diameter fitting the side cell to-wards SB following:								
- nominal implantationof 3.00 mm stent [9]	0.6	Ø	0.9	0.7	1.1	0.6–0.8 (for 2.75–3.50 mm device)	1.17 (for 5.0 mm stent)	Important for SB obstruction after cross-over stenting, ease of access to SB and final SB orifice area following balloon dilatation
- followed by over-expansion with 5 mm balloon [9]	1.5	Ø	1.7	2.1	1.6	1.5	Ø	
- followed by SB 3.0 mm nominal balloon inflation [9]	2.742	2.797	2.584	Ø	2.612	Ø	Ø	
SB obstruction % after POT-SB-rePOT sequence [26]	18.4	5.6	13.1	17.7	10	Ø	Ø	

LM, left main; MV, main vessel; SB, side branch; PCI, percutaneous coronary intervention; POT, proximal optimization technique; Ø, missing information.

In addition to metallic base and strut thickness, deliverability, scaffolding, and SB access are further impacted also by the construction method [12, 13]. Stents can be categorized as coil, slotted tube, or modular, depending on the construction method, with variability in trading, between radial force, flexibility, and SB access. While slotted tube stents are made from a metallic tube and then have the pattern cut out using laser etching, coil stents are built from wires that are wound into a circular coil, which allows more flexibility and deliverability but at the expense of less radial force and resistance to deformation. The modular stents are constructed using multiple repeat modules that are fused together to construct a stent tube [12]. Trade in platform flexibility-strength ratio can be further tunned with varying numbers of ring connectors and peaks in the crown, allowing the device to optimize favorable properties within same base design. As general rule, more connectors enhance the platform stability and integrity to deformation, while reducing the flexibility and side-cell opening area towards the SB. The structural differences among different DES, ultimately affect their performance and behavior during various steps of bifurcation PCI, as shown in Table 1.

## 4. Bifurcation Lesions

Bifurcation lesions, which have unique anatomical characteristics, put stent design to the ultimate test. Bifurcations in epicardial coronary arteries demonstrate a fractal pattern (a fractal is a geometric shape in which every smaller structure is similar to the whole part) [15]. With this geometry, the myocardium beneath is supplied with the optimal quantity of blood while consuming the smallest amount of energy. Simply said, coronary vessels narrow, but instead of tapering steadily, change in diameter happens abruptly following each branching. Thereby, a coronary bifurcation consists of a flow divider (carina) and three vessel segments with different diameters: The proximal MV, the distal MV and the SB. There is a constant relationship between these three vessels that was identified by Murray’s law a century ago as: ${\mathrm{(Diameter of proximal MV)}}^{7/3}$ = (${\mathrm{Diameter of distal MV)}}^{7/3}$ + ${\mathrm{(Diameter of SB)}}^{7/3}$ [16]. Finet’s formula adopted the equation according to intravascular ultrasound (IVUS) measurements in normal human coronary arteries: (diameter of proximal MV) = 0.678 (*i.e.*, approximately 2/3) × (diameter of distal MV + diameter of SB) [15]. Precisely, Huo-Kassab’s 7/3 model accurately predicts all size diameters of the epicardial coronary bifurcation vessels whereas Murray’s law and Finet’s formula can only do so in certain size subsets [17]. Finet’s formula is the one that is most frequently used in clinical practice in most Cath labs due to its ease of use.

**Fig. 3.**
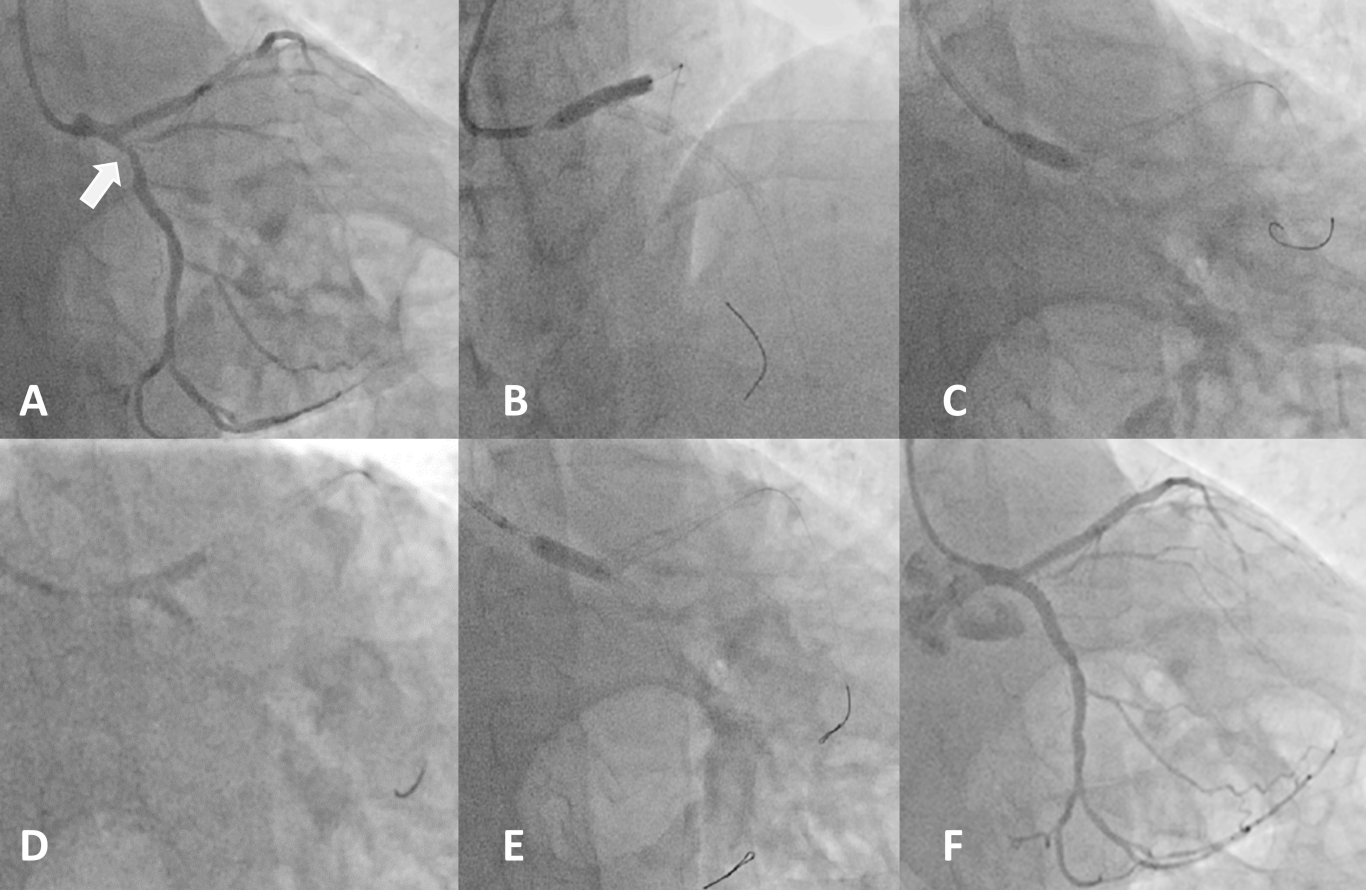
**Case example of LM bifurcation treated with inverted provisional stenting technique**. Panel (A) Ostial Cx lesion, bifurcation lesion Medina type 0.0.1 (white arrow). Panel (B) Implantation of DES (SYNERGY 4.0 × 24 mm) in cranial projection from proximal Cx up to the ostium of LM. Panel (C) POT with 5.0 × 12 mm balloon at high pressure. Panel (D) KBI with two 4.0 × 15 mm balloons at nominal pressure. Panel (E) Final POT. Panel (F) Final result. DES, drug eluting stents; Cx, circumflex artery; LM, left main; KBI, kissing balloon inflation; POT, proximal optimization technique.

**Fig. 4.**
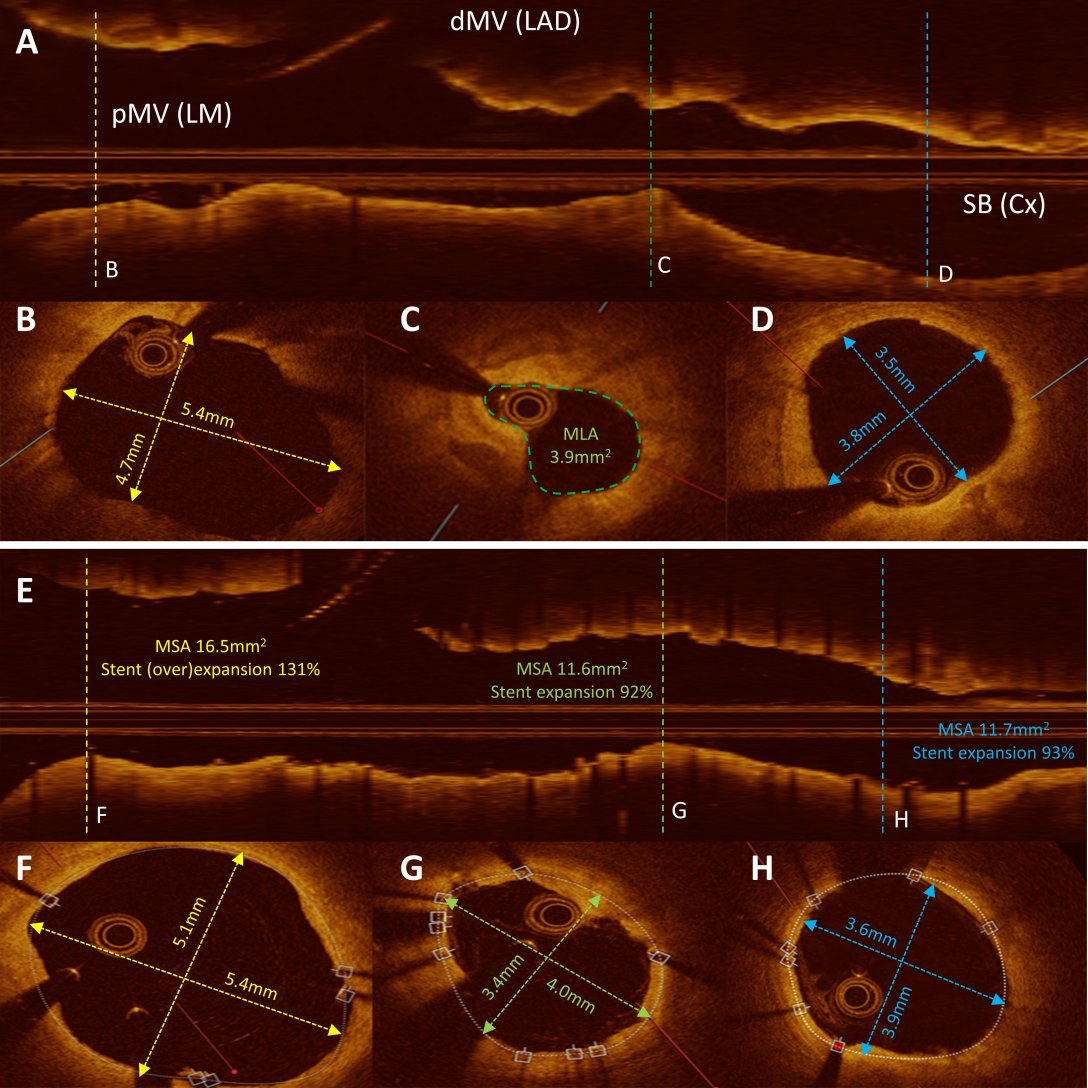
** Optical coherence tomography imaging showing pre and post intervention showing stent over-expansion in LM**. Panel (A) Longitudinal OCT reconstruction of the bifurcation. Panels (B) and (D) Cross section lumen measurements showing difference in diameters between pMV and SB (Cx) prior to PCI. Panel (C) Tight stenosis of near ostial Cx with MSA 3.9 ${\mathrm{mm}}^{2}$. Panel (E) Longitudinal OCT after PCI and stent implantation. Panels (F, G, and H) Cross section MSA measurement with stent expansion calculations. PCI, percutaneous coronary intervention; dMV, distal main vessel; LAD, left anterior descending; LM, left main; MLA, minimal lumen area; MSA, minimal stent area; OCT, optical coherence tomography; pMV, proximal main vessel; SB, side branch; Cx, circumflex artery.

Additionally, the fact that bifurcations have varying diameters in various patient subgroups, individuals, and different sites along coronary tree, further multiplies complexity. The average LM coronary artery diameter according to various measurements, reaches up to 4.75 mm, but substantial proportion of patients can have LM above 5 mm (up to 1/3 of patients) or even up to 6 mm, since anatomical variations in general population follow the rule of normal distribution [18, 19]. Males have larger coronary artery diameters than females, and ethnicity and age are prone to affect these differences. Further to this discrepancy, bifurcations also encompass region known as the polygon of confluence (POC), specific elliptically shaped area between the proximal, distal MV and SB whose boundaries are by convention formed by the lines drawn vertically in the ostium of branches and at the end of the proximal MV. Due to its size and shape, POC presents the frequent segment where marked strut malapposition can be found since stent needs to be stretched to its limits to be able to scaffold optimally contralateral sides to carina [20]. Finally, important aspect of every bifurcation is the so called “carinal angle” (angle between the distal MV and SB), since stent implantation in wider angle anatomy is related to increased rates of malapposition and can lead to stent fractures due to hinge motion, both, linked to adverse clinical events. Therefore, considering all specificities of the underlying anatomical substrate, the operator needs to predict the impact of the final stent configuration by careful selection of both, DES size and type, and stenting technique (Figs. 3,4) [21].

Further to this, reconstructing the bifurcation anatomy, avoiding stent malapposition and obtaining good scaffolding of all bifurcation segments, means deliberately altering the stent integrity and shape, frequently overcoming the manufacturers recommendations and labeled instructions for use. In a bifurcation stenting, most of the damage to the stent integrity comes from over-expansion and/or over-dilatation [3]. Over-expansion subjects the stent strut and the coating to extreme forces and deformation, increasing the risk of polymer coating damage, especially during maneuver specific to bifurcation PCI as proximal optimization technique (POT) with severe over-expansion or during kissing balloon technique (KB) (Figs. 3,4) [22]. Additionally, since coating damage can also occur during stent manipulations, special care should be exercised during device delivery, avoiding forceful maneuvers [23]. The polymer integrity and resistance to mechanical stress differ among different DES [24]. Therefore, it is advisable to choose a DES that has mechanically more resistant polymer, especially for complex bifurcation techniques. Operator unawareness of polymer integrity and possible mechanisms of polymer damage can lead to drug coating damage or detachment of debris that will expose patients to potential risks of thrombosis and inflammation with neointimal reactions, both related to worse prognosis with a greater degree of late lumen loss and restenosis rates [4, 22]. Further, stent over-expansion results in stent configuration with wider cells and larger separation of crowns, reducing the concentration of antirestenotic drugs intended to reduce neointimal proliferation, that may predispose DES restenosis [25].

On the other side, following POT, favorably as stent crowns straighten, the resulting scaffolding shape possesses greater radial force and resistance to acute recoil. But this also results in greater device stiffness due to the straightening of the crown close to the stent physical limit, which may increase risk of strut fracture due to metal fatigue on the stent [21]. Majority of the mentioned effects of stent accommodations to bifurcation specific anatomy became apparent following standardized *in-vitro* bench testing.

## 5. Bifurcation Bench Stent Testing

Concerns regarding stent behavior during PCI of bifurcation lesions led to an approach of pre- and post-marketing bench testing of the devices. Bench testing in general allows thorough stent or scaffold evaluation that include characteristics such as recoil, radial strength, flexibility, fracture resistance, longitudinal strength and security from dislodging from the delivery balloon with direct applicability of the findings [18]. For instance, during development of dedicated bifurcation stents need to be delivered over two wires, observations during bench delivery revealed “wire bias” caused by wire wrap as the main cause of delivery issues. Hence, according to this, an addition of a steerable shaft on a special bifurcation stent was provided for device placement, making it easier to unwrap wires overcoming the issue [18]. Additionally, a bench may indicate and identify procedural flaws like presence or absence of under-expansion, distortion, achieved cross-sectional area, diameters, eccentricity, and malapposition in stents or scaffolds. Specifically for bifurcation PCI, over-expansion phenomena like stent fracture or polymer coating damage can be investigated, as well as the proportion of the SB ostium that is occupied by jailing struts limiting access to branches [18]. In this regard, ease of SB access can be investigated assessing the delivery and integrity of various devices (stents or balloons) [9]. Even the most disrupting maneuvers, such as the KB inflations or stent crushing, can be performed to observe behavior of stents, all within patient-derived bifurcation anatomy [26].

## 6. Lessons from the Bifurcation Bench Testing

Bench test studies have provided important information that may be instrumental for carefully selecting the appropriate type and size of contemporary DES based on their design and stent behavior during test. Although *in-vitro* measurements may not perfectly mimic the mechanical behavior of stents *in vivo*, it offers reliable estimations that can assist operators in the process of device instrumentation decision-making. Bifurcation bench testing reports mainly focus on two most important bifurcation specific questions: over-expansion capability and side cell opening (Table 1). Due to the difference in lumen diameter between the bifurcation segments and the postulate that stent sizing is performed according to the distal MV diameter, POT has become obligatory step during any bifurcation stenting technique to ensure optimal stent apposition [1]. Choosing the device incapable to comply to the vessel reference diameter results in incomplete stent apposition, that has been associated with increased risk of in stent restenosis and stent thrombosis [27]. Based on bench data reports, we can state that most of the contemporary DES platforms:

- possess the capacity to considerably expand outside of their nominal diameter while maintaining their structural integrity (Table 1) [3, 28].

- over-expansion depends on stent design and stent size, varying between different platforms (from 25% to 75% higher than the nominal diameter, average 56%), but also within different sizes of the same DES type (large or small vessel design) [3, 28].

- over-expansion not only increases the minimal lumen diameter and stent area, but also increases the cell size due to straightening of the struts reaching up to 2.1 mm in circular diameter (important for SB access) (Table 1) [3, 28, 29].

- SB jailing ratio varies between different stent platforms ranging from 5.6% up to 18.7%, depending not only on the design but also on obtained configuration and unpredictable alignment of connectors and crowns (Table 1) [29].

- following SB balloon dilatation, the achieved stent cell circular fitting diameter is below the balloon diameter at nominal pressure (for 3 mm balloon maximal 2.7 mm); therefore, oversizing or overinflating can be considered especially in case of further SB stenting) (Table 1) [9].

- final side-cell diameter following balloon dilatation depends on number of connectors in a given DES platform (less connectors allow larger side-cell opening) [30].

- KB inflation causes stent overstretching in the proximal MV region when juxta positioning of the two KB is accomplished, resulting in higher eccentricity index, and greater number of malapposed struts irrespective of DES type [8, 20].

- final balloon optimization (i.e., repeat POT) ameliorates deleterious effects of KB inflation [8, 31].

According to the findings of bench testing, it is crucial to apprehend the stent’s expansion and overexpansion capabilities, as well as its ability to dilate SB cells regarding the specific underlying bifurcation anatomy and the treatment technique to be executed. Given that this information is not readily available, aside from standard compliance charts and burst pressure data, it must be acknowledged in advance and kept in mind by the operator during bifurcation PCI.

## 7. Computational Simulations of Mechanical Stent Performance

Alongside bench testing, advancements in computer science and technology provided new impactful research tool called computational stenting simulations (CSS). Precise predictions of stent behavior and performance in real case scenarios using patient-specific data and geometry can be performed with CSS. Overcoming the limitations of bench testing that lack lesion-like experience, CSS can even assess how different stent designs interact with various plaque materials in an patient-specific simulated environment [32]. Hypothetically, combining actual patient data and surrogate (non-clinical) endpoints (such as stent expansion, apposition, vessel scaffolding, side branch jailing, and fluid dynamics), results of CSS can even be extended to conduct in-virtual clinical trials predicting long-term clinical outcomes [33]. These benefits of CSS, especially if combined with *in-vitro* bench testing, open an entirely new perspective for the device industry, importantly, speeding up and streamlining the procedures for stent testing, development, and regulatory approval.

For example, optimal stent design in *in-vitro* and complementary CSS methods was suggested in the design process of a dedicated LM and large-sized arteries stent following a computational assessment of different designs of a new everolimus-eluting stent (SYNERGY MEGATRON, Boston Scientific Inc., Galway, Ireland) [34]. MEGATRON stent has been especially planned for the treatment of large proximal vessels, including LM bifurcations since it is optimized to provide high radial strength and overexpansion ability up to 6.0 mm, while maintaining vessel scaffolding of large vessels. During its development, three designs have been investigated and compared (9-peak, 10-peak, and 12-peak). The CSS suggested that 12-peak MEGATRON has enhanced vessel scaffolding, normalized hoop force/radial strength, and stent-to-artery ratio, as well as lesser vessel prolapse than the 10-peak and 9-peak designs. Based on these results and supplementary experimental bench testing data that confirmed the findings, the 12-peak stent design ultimately was considered optimal and became the commercially available version of the stent.

## 8. Clinical Data

With respect to clinical outcomes, no randomized trial directly compared the DES type one-to-another for this lesion subset, so objective preference cannot be given for a specific device type. Despite the absence of device-to-device comparative data, in September of 2022, the Food and Drug Administration (FDA) cleared Resolute Onyx Frontier (Medtronic, Minneapolis, MN, United States) as first DES to receive indication for non-LM bifurcation PCI based on data from the Resolute Bifurcation Study [35]. Resolute Onyx Frontier DES demonstrated low event rates, achieving the performance goal for the primary endpoint of target vessel failure (TVF) at one year [36]. In a total of 205 patients with 207 bifurcation lesions among which 32.4% of lesions were classified to be true bifurcation lesions with disease of the SB, the rate of the primary endpoint of TVF at 1 year was 6.9% with a 1-sided upper 95% confidence interval of 10.5%, significantly lower than the pre-specified performance goal (*p* <0.001). At 1-year, cardiac death was 1.5%, clinically driven target vessel revascularization 3.4%, and target vessel myocardial infarction (MI) 2.9%. There were no cases of definite/probable stent thrombosis.

Accumulated data from clinical trials, bench tests, and CSS, lead to the advancements in stent design in general, resulting in net clinical advantages of newer over earlier device iterations. Specifically, a propensity score matched study with a population of 5489 patients compared the efficacy and safety of first- versus second-generation DES at the 5-year follow-up in patients who underwent bifurcation PCI from COBIS (Coronary Bifurcation Stenting) registries II and III [37]. Five-year target lesion failure (TLF) (the composite of cardiac death, MI, and target lesion revascularization (TLR) and cardiac death or MI were compared between the use of first-generation DES, n = 2436) and second-generation DES (n = 3062) during PCI. Patients treated with second-generation DES had a significantly lower risk of TLF at 5 years than those treated with first-generation DES in both overall and propensity-matched populations (matched hazard ratio [HR matched]: 0.576; 95% confidence interval [CI]: 0.456 to 0.727; *p* < 0.001). Overall, the risk of cardiac death or MI did not differ between the first- and second-generation DES era. However, the use of second-generation DES was associated with a significantly lower risk of cardiac death or MI in patients who required a 2-stent technique for a bifurcation lesion.

## 9. LM Bifurcation — Anatomical, Procedural, and Clinical Considerations

About 5% of patients having coronary angiography will have LM disease, and typically this condition is associated with severe downstream coronary artery disease, and indication for myocardial surgical revascularization for these patients [38]. Recently, randomized trials proved that PCI can provide equivalent results in this patient subset with less extensive downstream disease, using contemporary DES and optimal stenting strategy [39]. Although comparable to other non-LM lesions, the anatomical specificities and clinical importance of the LM bifurcation necessitate highlighting and taking into account during PCI with DES: (1) LM supplies roughly 70% of the myocardial mass overall, and any procedural compromise can have immediate negative consequences; (2) Cx is typically considered as SB; however, due to its large diameter (average 3.2 ± 0.7 mm) and clinical significance, the idea of SB needs to be regarded relatively so; (3) the average LM diameter is between 4 and 5 mm (4.2–4.75 mm on intravascular studies), and maximum expansion ability of available stents should be thus considered carefully, especially if using stents ≤3 mm (i.e., during inverted provisional); (4) Additional ramifications, trifurcations or quadfurcations, are relatively common (in 10% to 15% of cases). As a result, multi-balloon simultaneous KB, or even “trissing”, triple concomitant balloon inflation, can be required and may result in significant stress and morphological changes to the stent platform; (5) The bifurcation angle between the left anterior descending (LAD) and Cx is wider, ranging from 72 to 96 degrees, than non-LM bifurcations, which is typically 46 to 64 degrees. This wider angulation predisposes to shorter fatigue life of stent platforms with fracture risk [18].

On top of these specificities, clinical results of LM bifurcation stenting are worse compared to non-LM bifurcations [40]. Recently reported large registry data showed that patients treated with PCI for an LM bifurcation had poorer outcomes than those with a non-LM bifurcation in the second-generation DES era, irrespective of the stent design (TLR, hazard ratio (HR) adjusted, 1.846 [95% CI, 1.317–2.588]; *p* < 0.001). Only for the LM bifurcation group, compared with the 1-stent strategy, the 2-stent strategy showed a significantly higher risk of TVF (2-stent versus 1-stent, 17.4% versus 10.6%; HR adjusted, 1.848 [95% CI, 1.045–3.266]; *p* = 0.035), mainly driven by the higher rate of TLR (15.3% versus 5.5%; HR adjusted, 2.698 [95% CI, 1.276–5.706]; *p* = 0.009). This further strengthens the current recommendations from the European Bifurcation Club (EBC), that provisional single stent strategy should be preferred strategy [1, 6]. In addition, it highlights the need for optimizing the procedural results of LM bifurcation stenting, preferably using IVUS guidance, especially in circumstances that mandate complex 2-stent strategy [41].

Considering the unique anatomical characteristic and worse clinical outcomes of LM bifurcation PCI compared to non-LM procedure, emergence of specifically designed device such as SYNERGY MEGATRON, according to first reports, provided to interventional cardiologists stent properties that can facilitate optimal stent implantation [42]. In a recent report, in 98 patients treated with this novel stent, optimal stent implantation was achieved in 88% of the cases, using minimal stent area (MSA) >90% compared to proximal reference as criterion for the LM region. Obtained final MSA in LM in this population of 14.5 ± 3.4 ${\mathrm{mm}}^{2}$ were clearly above the 12.5 ± 3.0 and 9.9 ± 2.3 ${\mathrm{mm}}^{2}$ MSA that were reported for two largest randomized LM trials, NOBLE and EXCEL, respectively [43, 44]. Contrary to LM, measured ostial MSA for left anterior descending (10.0 ± 2.5 vs. 10.1 ± 2.9 ${\mathrm{mm}}^{2}$) and left circumflex artery (9.8 ± 3.0 ${\mathrm{mm}}^{2}$ vs. 9.6 ± 3.4 ${\mathrm{mm}}^{2}$) were comparable. This illustrates the new stent platform’s ability to over-expand, even up to 6 mm with a 3.5 mm stent platform, when compared to devices utilized in earlier trials.

## 10. Dedicated Bifurcation Stents

Because conventional stents are not specifically made for bifurcation PCI, considering the specific anatomy, requirement for continuous access to the SB, irregular device overlapping and strut distributions, all being dependent on technique and device used, dedicated bifurcation stents (DBS) have been developed to tackle these issues. They were introduced with bifurcation-specific engineering advancements for technically simple and high procedural success rates, while safeguarding the SB by allowing permanent or unchallenged SB access as well as providing optimal main branch (MB) and SB scaffolding and coverage, limiting the use of multiple layers of stent struts, without gaps in scaffolding, with an ultimate goal to translate this in optimized short- and long-term results [45]. Based on their primary bifurcation segment target, they can be divided in two groups: (1) main vessel DBS (MV-DBS) dedicated to treatment of the MV, that facilitate or maintain access to the SB, and (2) side branch DBS (SB-DBS), dedicated to treating and protecting the SB first. MV-DBS include the following devices: AxxessTM (Biosensors International, Singapore, Singapore), BiOSS Expert and BiOSS LIM (Balton, Warsaw, Poland), Nile CroCo and Nile PAX (Minvasys, Gennevilliers, France), STENTYSTM (STENTYS SAS, Paris, France), Xience SBATM (Abbott Vascular, Santa Clara, CA, USA), Twin RailTM (Invatec/Medtronic, Roncadelle BS, Italy), TAXUS PetalTM (Boston Scientific, Marlborough, MA, USA) and others. Those stents allow placement of a second stent in SB branch if needed as during provisional approach. In most MV-DBS, the SB opening is located at the center of a stent and the proximal part of side branch balloon is mounted within the main branch stent. Contrary to expectations, most MV-DBS require extensive operator and device experience, and have device-specific technical issues such as wire wrapping or twisting during delivery, difficult system torque control and predictive alignment (both axial and rotational) with the SB ostium, that preclude them from being easily widely adopted.

Although many types are available, 4 DBSs were studied in randomized trials: BiOSS Expert and BiOSS LIM, the Tryton stent (Tryton Medical, Durham, NC, United States) and the Axxess bifurcation stent. The BiOSS Expert is a paclitaxel-eluting balloon-expandable dedicated bifurcation stent that is implanted in the MV and with an open side access to the ostium of the SB [45]. The BiOSS LIM is a sirolimus-eluting balloon-expandable dedicated stent. Both, devices are designed to respect the fractal geometry of bifurcation, hence the proximal region has a larger diameter than the distal (the proximal/distal diameter ratio is 1.15–1.3) while being mounted on a special stepped diameter delivery balloon. The Tryton stent is a balloon expandable dedicated cobalt chromium non-DES, being most widely studied device among SB-DBS. This stent is implanted in the SB, and a DES is implanted in the MV through the large open struts design at the POC level of this dedicated stent. The Axxess stent is a self-expandable biolimus-eluting dedicated stent that is implanted in the proximal MV with its distal end aligned to the carina, allowing easy access to both the distal MV and the SB, and additional stent implantation for distal vessels, if required (80.9% of patients in AXXES Plus pivotal study) [46].

Unfortunately, despite their technical and distinctive design qualities suited for bifurcation PCI, none of the DBSs have yet demonstrated better clinical results than conventional DES when used for a stepwise, layered provisional bifurcation stenting strategy, as outlined by the European bifurcation club [1, 6, 45, 47].

## 11. Conclusions

In conclusion, treating coronary bifurcation lesions with PCI and DES presents unique challenges, requiring careful consideration of bifurcation anatomy, operators experience, stenting strategy and stent design characteristics. Matching the proximal MV diameter and providing easy access to the SB are critical for bifurcation stenting success. In order to accomplish optimal stent expansion and apposition, operators must be aware of stent design properties, such as maximal expansion capacity and side-cell opening towards the SB. Bench testing and CSS present valuable tools in stent design process, pre- and post-marketing evaluation, and optimization of bifurcation stenting strategies. Important information regarding device characteristics and procedural behavior facilitates further device development, and guide clinicians in procedure planning and optimal DES selection. Data obtained by experimental methods, hands-on operator feedback and clinical results of real-world populations, must be synthesized in order to make a proper DES selection aiming to improve procedural success and patient outcomes in this complex lesion subset.
